# Acceptance as a Mediator for Change in Acceptance and Commitment Therapy for Persons with Chronic Pain?

**DOI:** 10.1007/s12529-015-9494-y

**Published:** 2015-06-04

**Authors:** Jenny Thorsell Cederberg, Martin Cernvall, JoAnne Dahl, Louise von Essen, Gustaf Ljungman

**Affiliations:** Department of Women’s and Children’s Health, Uppsala University, 751 85 Uppsala, Sweden; Clinical Psychology in Healthcare, Department of Public Health and Caring Sciences, Uppsala University, Uppsala, Sweden; Department of Psychology, Uppsala University, Uppsala, Sweden

**Keywords:** Acceptance, Acceptance and commitment therapy, Chronic pain, Mediation analysis, Physical functioning, Satisfaction with life

## Abstract

**Background:**

Cognitive behavior therapy (CBT) is considered effective for chronic pain, but little is known about active treatment components. Although acceptance correlates with better health outcomes in chronic pain patients, no study has examined its mediating effect in an experimental design.

**Purpose:**

The aim of the present study is to investigate acceptance as a mediator in acceptance and commitment therapy (ACT), a third wave CBT intervention, for chronic pain.

**Method:**

A bootstrapped cross product of coefficients approach was used on data from a previously published RCT evaluating ACT for chronic pain. To address the specificity of acceptance as a mediator, anxiety and depression were also tested as mediators. Outcome variables were satisfaction with life and physical functioning. Two change scores, pre-assessment to 6-month follow-up (*n* = 53) and pre-assessment to 12-month follow-up (*n* = 32), were used.

**Results:**

Acceptance was found to mediate the effect of treatment on change in physical functioning from pre-assessment to follow-up at 6 months. Further, a trend was shown from pre-assessment to follow-up at 12 months. No indirect effect of treatment via acceptance was found for change in satisfaction with life.

**Conclusion:**

This study adds to a small but growing body of research using mediation analysis to investigate mediating factors in the treatment of chronic pain. In summary, the results suggest that acceptance may have a mediating effect on change in physical functioning in ACT for persons with chronic pain. However, given the small sample size of the study, these findings need to be replicated.

## Introduction

According to the World Health Organization (WHO), chronic pain is one of the most underestimated challenges for health care worldwide [1]. In chronic pain, comorbidity with depression and anxiety is common [2–4], social life is negatively affected [5], daily functioning impaired [6–8], and general level of activity reduced [3, 9]. On the whole, chronic pain reduces quality of life for the patient [10–13] and imposes high expenses on health care systems [1]. Cognitive behavior therapy (CBT) has been applied for chronic pain for decades with positive outcomes [14]. The definition of CBT is, however, broad, and the strategies applied within CBT for chronic pain may include a wide range of therapeutic tools [15]. This lack of specificity creates uncertainty as to the processes at work in CBT treatment [16]. A recent Cochrane review concludes that CBT is useful for chronic pain and that there is no need for further RCTs focusing on the reporting of group mean values [17]. Instead, studies identifying effective components of treatment are requested. Acceptance and commitment therapy (ACT), developed within the third wave of CBT, shares important features with CBT but is derived from functional contextualism and relational frame theory and thus has distinct philosophical and theoretical assumptions [18]. ACT has been shown to improve mental and physical health [19] and has been listed by the American Psychological Association (APA) as having strong research support for chronic pain [20]. In ACT, the aim is to create psychological flexibility around impairing life experiences, such as chronic pain, to enable moving forward and engaging in a vital valued life [21]. Psychological flexibility includes several processes, of which acceptance is one. Acceptance of chronic pain is defined as living with pain without reacting to, judging or attempting to reduce or avoid it [22]. It is not resignation to or ignoring pain but rather an active willingness to engage in meaningful activities in the presence of pain. Psychological acceptance has been shown to be beneficial in chronic pain [23–28] although most of the data is cross-sectional and/or correlational, which may establish covariation between variables but does not allow causal inferences [29, 30]. In two studies by McCracken and Gutiérrez-Martínez [31] and Vowles and colleagues [32], acceptance was shown to correlate with positive changes in disability, depression, and pain-related anxiety. Both of these studies, however, lacked experimental design. Randomized controlled trials (RCTs) testing the mediating effect in treatments for chronic pain are very sparse. In two RCTs by Wicksell and colleagues [33, 34], psychological flexibility was shown to mediate the effect of ACT on depression, pain-related functioning, and life satisfaction. Psychological flexibility is, however, comprised in the ACT model by acceptance as well as other therapeutic processes.

In summary, research on the mechanisms of change in ACT for chronic pain is scarce. Acceptance is a key concept in the theoretical model, but there is no experimental study investigating its mediating effect. The aim of this study is to investigate whether acceptance mediates the effect of treatment on satisfaction with life and physical functioning using data from a previously published RCT evaluating ACT for chronic pain [35].

## Method

### Setting and Participants

**Fig. 1:**
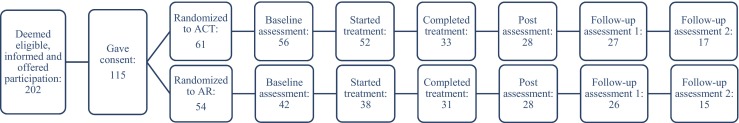
The participants’ flow in the main study [35]

Participants, with all types of chronic pain excluding malignancies, were recruited from the Pain Center at Uppsala University Hospital. Beyond a diagnosis of chronic pain, inclusion criteria included being accessible for treatment during a 7-week period and sufficient literacy skills in Swedish to be able to follow the treatment manual. Two hundred and two patients were deemed eligible and offered participation in the study. Of these, 115 gave written informed consent and were randomized to either ACT or applied relaxation (AR). Ninety participants started treatment. Of these, 64 participants completed the treatment, 56 completed post-assessment, and 53 and 32 participants, respectively, completed follow-up assessments at 6 and 12 months (see Fig. 1 for participants’ flow). The participants’ mean age at study start was 46.0 years (SD = 12.3), 36 % were men and 64 % women. The majority (98 %) reported having had pain for more than 1 year, 62 % were on sick-leave, 27 % were working or studying part- or full-time, and 8 % were retired. The study was approved by the Regional Ethical Committee in Uppsala, Sweden.

### Interventions

Both interventions, ACT and AR, were manual-based self-help treatments with weekly therapist support via the telephone. The duration of both interventions was 7 weeks, with an initial and a concluding 90-min session in vivo. During the self-help phase of treatment, participants worked with assigned chapters of the treatment manual with scheduled weekly 30-min telephone sessions. Guided self-help has been shown to be equally effective as face-to-face treatment for depression and anxiety [36]. Furthermore, telephone administered CBT has shown comparable clinical efficacy compared to face-to-face treatment for depression among primary care patients [37] and in multiple sclerosis [38]. Participants also had the opportunity to e-mail their therapist for support if necessary throughout the treatment. The interventions are described below. Note that the focus in this study is on the evaluation of ACT. AR has previously been evaluated for chronic pain [39, 40] and functions in this case as an active treatment control group.

### Acceptance and Commitment Therapy

The initial face-to-face session of the ACT intervention consisted of the mapping of pain strategies in relation to short- and long-term goals, as well as the identification of values and to what extent the participant was living in accordance with these. The session concluded with an introduction to a Swedish version of the treatment manual *Living beyond your pain* [41]. During the self-help phase, the participants worked through the treatment manual covering perspective-taking on own thoughts and self-conceptions, mindfulness and acceptance strategies, identification of obstacles to living in accordance with personal values, and formulation of a committed action plan (for a detailed description of the content of each chapter of the treatment manual, see Dahl and Lundgren [41] or Table 1 in Thorsell et al. [35]). During the weekly telephone sessions, the topic for the week was discussed. The concluding face-to-face session consisted of a discussion about the participant’s values, obstacles, and plan for action to engage in meaningful life activities.

### Applied Relaxation

The initial face-to-face session of the AR intervention consisted of the mapping of challenging pain situations and a discussion about AR as a coping method as well as a preventive strategy. Further, the session consisted of a practical introduction to the method and an introduction to the treatment manual, a self-help version of the original AR manual [42]. During the self-help phase, the participants worked through the treatment manual, consisting of the following steps with a gradual increase in level of difficulty: differentiation between tension and relaxation, cue-controlled (self-instructed) relaxation, application of relaxation to different settings, rapid relaxation, and application of relaxation to everyday life activities including stressful situations (for a detailed description of the content of each step of the treatment manual, see Table 1 in Thorsell et al. [35]). During the weekly telephone sessions, the practical application of the week was discussed. The concluding face-to-face session consisted of a discussion about how to maintain the acquired skills and formulation of a maintenance program.

### Measures

Measures were taken at four time points. Pre-assessment was carried out 1 to 2 weeks prior to start of intervention and post-assessment at the end of treatment. Follow-up assessments took place at 6 months (follow-up 1) and 12 months (follow-up 2) after completion of treatment. Variables measured for relevance for the current study are as follows: acceptance of chronic pain, satisfaction with life, physical functioning, anxiety, depression, and pain intensity.

### Acceptance of Chronic Pain

Acceptance was measured by the Chronic Pain Acceptance Questionnaire (CPAQ) [43], entailing two subscales. The Activities Engagement scale (11 items) measures engagement in meaningful activities in the presence of pain. The Pain Willingness scale (nine items) measures willingness to experience pain and the degree to which the respondent tries to avoid or control pain. Items are rated on a scale from 0 = “never true” to 6 = “always true.” Some items are reversed, and high scores indicate a high level of acceptance. Internal consistency has been shown to be 0.78 to 0.82, and the scale correlates negatively with measures of physical disability and psychological ill health [43].

### Satisfaction with Life

Satisfaction with life was measured by the Satisfaction with Life Scale (SWLS) [44], measuring general satisfaction with life. The SWLS contains five statements, e.g., “In most ways my life is close to ideal,” that the respondents are asked to agree or disagree with on a scale from 1 to 7. High scores indicate satisfaction whereas low scores indicate dissatisfaction with life. The scale has good internal consistency (α = 0.88 in a Swedish trial [45]) and validity and has been shown to be sensitive to change [46, 47].

### Physical Functioning

Physical functioning was measured by five items of the Örebro Musculoskeletal Pain Questionnaire (ÖMPQ) [48], for which factor structure and psychometric properties have been supported [49–51]. The respondents rate their ability to carry out light work and household chores, walk for an hour, shop for groceries and sleep on a scale from 0 (“cannot do at all due to pain”) to 10 (“can do without pain problems”). High scores indicate a high level of physical functioning.

### Anxiety and Depression

Anxiety and depression were measured by the Hospital Anxiety and Depression Scale (HADS) [52] containing two subscales with seven items each, 14 in total. The scale consists of statements that the respondents rate on a scale from 0 to 3. Higher scores indicate higher levels of anxiety and/or depression. The internal consistency in a Swedish study was 0.84 for the anxiety subscale and 0.82 for the depression subscale [53].

### Pain Intensity

Pain intensity during the last week was measured by an NRS scale where the respondents rate their level of pain from 0 (“no pain at all”) to 10 (“unbearably lot of pain”); thus, high scores indicate a high level of pain. The NRS scale for measuring pain has good reliability and validity and has been shown to be sensitive to change [54, 55].

### Summary of the Results from the RCT

There was a significant condition by time effect in acceptance, where the ACT group reported increased acceptance from pre-assessment to post-assessment and from pre-assessment to both follow-up assessments while the AR group did not report any changes in acceptance. Regarding satisfaction with life, there was a significant effect of time and a trend toward a condition by time effect. The ACT group reported improvement from pre-assessment to post-assessment and to both follow-up assessments while the AR group did not report any changes in satisfaction with life. Regarding physical functioning, there was a significant condition effect, where the ACT group reported improvement from pre-assessment to post-assessment and from pre-assessment to follow-up 1 while the AR group did not report any improvement in physical functioning. Regarding anxiety and depression, there were significant time effects, where both groups improved. The ACT group reported decreased anxiety from pre-assessment to post-assessment and to both follow-up assessments and decreased depression from pre-assessment to post-assessment and to follow-up 2. The AR group reported decreased anxiety and depression from pre-assessment to follow-up 2. Regarding pain intensity, there was a significant condition effect where the ACT group reported a decrease from pre-assessment to post-assessment and from pre-assessment to follow-up 2 while the AR group did not report any decrease in pain intensity (see Thorsell et al. [35] for a detailed presentation of the results from the RCT).

### Statistical Analyses

All statistical analyses were performed in IBM SPSS Statistics, version 20 [56]. Statistical significance was interpreted conventionally, with *p* < 0.05 as “significant” and with *p* < 0.10 as indicating a trend.

### Preliminary Analyses

Descriptive statistics were carried out to provide an overview of the mean and change scores.

### Tests of Indirect Effects

**Fig. 2:**
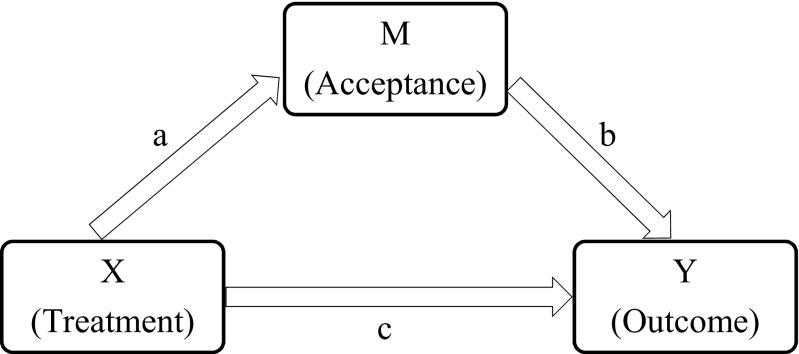
The mediation model

In mediation analysis, the indirect effect of variable *X* on outcome in variable *Y* via one (or more) mediator variable(s) *M* is investigated [57, 58] (see Fig. 2). There are a number of different methods to test mediation [59, 60]. The most widely used is *the causal steps* method [29, 57], which focuses on the individual paths of the model. The *product of coefficients* approach computes the product of the *ab* path, assessing the indirect effect of *X* on *Y* through *M* directly [61, 62]. It is a more powerful way to test mediation which requires only the presence of an effect to be mediated and that the indirect effect runs in the direction proposed by the mediation hypothesis. Note that a statistically significant total effect of *X* on *Y* is not necessary for mediation to occur and that mediation analysis does not require evidence of a total effect prior to investigating direct and indirect effects [63–66]. In the product of coefficients approach, the product distribution often violates the assumption of normal distribution, especially in smaller samples. *Bootstrapping* is a nonparametric procedure that acknowledges this fact [61, 62]. In bootstrapping, a large number of samples is taken (with the original sample size) from the data and the indirect effect, *ab*, is computed for each sample. The point estimate of *ab* is the mean *ab* computed from all samples while the estimated standard error is the standard deviation of all *ab* estimates of the samples. Confidence intervals (CIs) are derived from sorting the *ab* estimates from low to high. If the lower and upper bounds of the CI do not include zero, the indirect effect is significant. Simple mediation analyses were carried out using the PROCESS Syntax procedure for SPSS developed by Hayes [30]. In all bootstrap analyses 10,000 samples were used. No imputation was utilized. The analyses were performed with bias-corrected CIs of 95 and 90 %. Indirect effects with confidence intervals not including zero at CI = 95were interpreted as statistically significant and as a trend at CI = 90.Treatment was analyzed as the independent variable, with two levels: ACT and AR. Post-assessment acceptance scores were analyzed as mediator variable and changes in satisfaction with life and physical functioning as outcome variables. In order to assess the specificity of acceptance as a mediator, anxiety and depression at post-assessment were also tested as mediators. Two change scores were used for each outcome variable: change from pre-assessment to follow-ups 1 and 2. Indirect effects are reported as unstandardized estimates.

### Supplementary Analyses

Two steps of supplementary analyses were carried out. Firstly, indirect effects were tested including potential covariates. This procedure allows investigation of the indirect effect of treatment via the mediator on change in the outcome variable(s) while controlling for other variables. Change in pain intensity from pre-assessment to post-assessment and post-assessment score in the outcome were used as covariates. Secondly, a series of hierarchical multiple regression (HMR) analyses were carried out to investigate treatment-specific effects (allowing detailed comparisons between groups) and to provide information about the unique contribution of the predictor variables.

## Results

### Preliminary Analyses

Mean scores for the mediator and outcome variables from all the assessments are presented in Table 1. Mean change scores from pre-assessment to follow-up assessment on satisfaction with life and physical functioning are presented in Table 2.

**Table 1:** Mean scores (standard deviation) for all assessments

	Mean score (standard deviation)								
	Pre (*n* = 90)		Post (*n* = 56)		Follow-up 1 (*n* = 53)		Follow-up 2 (*n* = 32)		Scale range
	ACT	AR	ACT	AR	ACT	AR	ACT	AR	
Acceptance	47.52 (16.92)	46.97 (14.71)	62.48 (18.70)	51.78 (18.96)	57.85 (19.62)	53.35 (18.94)	60.82 (15.15)	50.53 (23.26)	0–120
Anxiety	9.15 (4.57)	8.11 (4.90)	7.61 (4.58)	7.37 (5.25)	8.52 (4.59)	7.15 (4.91)	6.41 (3.43)	7.53 (4.19)	0–21
Depression	8.69 (4.48)	8.95 (4.26)	6.64 (4.61)	7.33 (4.81)	7.04 (4.80)	7.96 (5.38)	7.18 (5.28)	7.53 (4.69)	0–21
Satisfaction with life	16.96 (6.46)	16.87 (6.82)	21.48 (7.07)	18.04 (7.07)	18.89 (6.41)	16.58 (7.23)	21.06 (6.07)	16.53 (8.37)	0–35
Physical functioning	5.32 (2.23)	4.61 (2.17)	6.24 (2.33)	4.81 (2.49)	6.32 (2.23)	4.87 (2.65)	6.29 (2.37)	5.13 (3.27)	0–11
Pain intensity	7.94 (1.63)	8.34 (1.74)	7.21 (1.95)	7.86 (2.09)	7.63 (1.88)	7.84 (1.97)	7.00 (2.25)	8.40 (2.17)	0–11

Follow-up 1 = 6 months; follow-up 2 = 12 months

*ACT* acceptance and commitment therapy, *AR* applied relaxation

**Table 2:** Mean change scores (standard deviation) for satisfaction with life and physical functioning from pre-assessment to follow-up assessment

	Mean change score (standard deviation)			
	Pre-assessment to follow-up 1 (*n* = 53)		Pre-assessment to follow-up 2 (*n* = 32)	
	Satisfaction with life	Physical functioning	Satisfaction with life	Physical functioning
ACT	3.26 (5.27)	0.70 (1.97)	2.24 (7.52)	0.66 (2.39)
AR	−0.31 (5.61)	0.26 (1.31)	0.07 (5.55)	−0.28 (2.05)

Follow-up 1 = 6 months; follow-up 2 = 12 months

*ACT* acceptance and commitment therapy, *AR* applied relaxation

### Tests of Indirect Effects

Results from the mediation analysis are presented in Table 3.

**Table 3:** Results from the mediation analyses with change in satisfaction with life and physical functioning from pre-assessment to follow-up assessment as outcome variables and acceptance of chronic pain, anxiety, and depression at post-assessment as mediator variables

Outcome (change score)	Mediator	Indirect effect	Bootstrap results for indirect effects			
			95 % CI		90 % CI	
			Lower	Upper	Lower	Upper
Satisfaction with life						
Pre to follow-up 1	Acceptance	0.339	−0.582	2.609	−0.378	2.175
	Anxiety	−0.001	−0.601	0.512	−0.409	0.367
	Depression	0.087	−0.353	1.505	−0.252	1.181
Pre to follow-up 2	Acceptance	0.893	−1.421	6.260	−1.036	5.228
	Anxiety	0.051	−1.327	1.628	−1.009	1.229
	Depression	0.179	−1.153	3.644	−0.859	2.869
Physical functioning						
Pre to follow-up 1	Acceptance	0.331	*0.005*	*1.021*	–	–
	Anxiety	−0.029	−0.376	0.129	−0.287	0.085
	Depression	0.107	−0.119	0.519	−0.068	0.452
Pre to follow-up 2	Acceptance	0.683	−0.005	2.122	*0.089*	*1.827*
	Anxiety	0.184	−0.199	1.079	−0.145	0.888
	Depression	0.203	−0.249	1.704	−0.142	1.322

Number of bootstrap samples = 10,000. The indirect effect is statistically significant at the 95 % confidence interval (CI) and marginally significant at the 90 % CI, when the CI does not include 0. Follow-up 1 = 6 months; follow-up 2 = 12 months; *n* = 43 in pre-assessment to follow-up 1; *n* = 27 in pre-assessment to follow-up 2

### Satisfaction with Life

No indirect effect of treatment via any of the mediators was found on change in satisfaction with life from pre-assessment to either follow-up assessment.

### Physical Functioning

A statistically significant indirect effect of treatment via acceptance was found on change in physical functioning from pre-assessment to follow-up 1. A trend toward an indirect effect of treatment via acceptance was found from pre-assessment to follow-up 2. No indirect effects of treatment via anxiety or depression were found from pre-assessment to either follow-up assessment.

### Supplementary Analyses

#### Physical Functioning

As seen in Table 4, when controlling for change in pain intensity, there was a trend toward an indirect effect of treatment via acceptance on change in physical functioning from pre-assessment to follow-up 1. According to the HMR analysis, the addition of acceptance in explained variance in change from pre-assessment to follow-up 1 was 17 % (∆*F* = 4.21, *p* = 0.05) for the ACT group. The indirect effect of treatment via acceptance on change from pre-assessment to follow-up 2 was statistically significant. The HMR analysis showed that acceptance explained an additional 26 % of the variance in change in physical functioning for the ACT group after adjusting for change in pain intensity (∆*F* = 3.58, *p* = 0.09).

**Table 4:** Results from the mediation analyses including change in pain intensity and physical functioning at post-assessment as covariates, change in physical functioning from pre-assessment to follow-up as outcomes, and acceptance of chronic pain at post-assessment as mediator variable

Covariate	Outcome	Indirect effect—acceptance as mediator	Bootstrap results for indirect effects controlling for covariate variables			
	Physical functioning		95 % CI		90 % CI	
			Lower	Upper	Lower	Upper
Pain intensity	Pre to follow-up 1	0.242	−0.016	0.959	*0.008*	*0.824*
	Pre to follow-up 2	0.946	*0.116*	*2.579*	–	–
Physical functioning at post-assessment	Pre to follow-up 1	0.199	−0.020	0.773	*0.003*	*0.680*
	Pre to follow-up 2	0.836	−0.036	2.771	*0.091*	*2.346*

Number of bootstrap samples = 10,000. The indirect effect is statistically significant at the 95 % confidence interval (CI) and marginally significant at the 90 % CI, when the CI does not include 0. Follow-up 1 = 6 months; follow-up 2 = 12 months; *n* = 43 in pre-assessment to follow-up 1; *n* = 27 in pre-assessment to follow-up 2

Further, as seen in Table 4, when controlling for earlier change in physical functioning, there were trends toward indirect effects of treatment via acceptance on change from pre-assessment to both follow-up assessments. The HMR showed that acceptance made a significant contribution of 35 % to follow-up 2 (*∆F* = 5.43, *p* = 0.04).

## Discussion

The results showed no indirect effect of treatment via acceptance on change in satisfaction with life from pre-assessment to either follow-up. There was, however, a statistically significant indirect effect on change in physical functioning from pre-assessment to follow-up 1 and a trend toward an indirect effect from pre-assessment to follow-up 2.Trends are reported in the current study due to the lack of research on the mediating effect of acceptance in chronic pain and the exploratory nature of the study with a small sample size.

There are no available power calculations for mediation analysis, but empirical data recommends different sample sizes depending on the strength of the association of the α- and β-path [67]. In the light of that data, the current study has relatively low power, which may be part of the explanation for the lack of support for an indirect effect of treatment via acceptance on change in satisfaction with life.

As to physical functioning, when adjusting for change in pain intensity, there was a trend toward an indirect effect on change from pre-assessment to follow-up 1 and a statistically significant indirect effect on change from pre-assessment to follow-up 2 (where acceptance explained an additional fourth of the variance). This suggests that the change in physical functioning is not merely ascribable to lower levels of pain but rather to higher levels of acceptance. Acceptance involves a perspective on pain where simultaneous engagement in valued activities is made possible. Engagement in activities which have previously been avoided naturally increases the level of physical functioning. When adjusting for earlier change in physical functioning, there were trends toward indirect effects to both follow-ups. The fact that the indirect effects were not significant at the 95 % CI raises questions regarding the temporal relation between the variables [68]. More specifically, whether acceptance is the mediating variable for changes in physical functioning or if level of function started to change before the level of acceptance did. It could be possible that physical functioning mediates changes in acceptance. Post-assessment scores on acceptance were used as the mediator variable. Continuous assessments during the course of treatment would have added important information in investigating the process of change in the relevant variables. With that in mind, there were still trends of indirect effects of treatment via acceptance on change in physical functioning after adjusting for earlier change in physical functioning. The HMR analysis further showed that acceptance made a significant additional contribution of 35 % in explained variance at follow-up 2 after adjusting for earlier change in physical functioning. This suggests that acceptance does mediate the effect of treatment on physical functioning.

Anxiety and depression were included as mediators in the analysis in order to address the specificity criterion. The fact that neither anxiety nor depression mediated the effect in physical functioning while acceptance did strengthens the case for acceptance as a mediating factor in the treatment.

Altogether, these results are in line with previous research [31, 32] suggesting that acceptance mediates the effect of ACT on change in physical functioning, and thus is a relevant treatment component, for people suffering from chronic pain.

The amount of attrition is a limitation since it reduces the power of the study. Considering the increases in acceptance in the ACT group alongside the improvements in the outcome variables, the nonsignificant indirect effect on change in satisfaction with life, as well as the trends regarding physical functioning, might have reached statistical significance had more participants been retained in the study. Regarding generalizability, the attrition is not considered as problematic. High rates of attrition are to be expected in bibliotherapy [69] and it could be argued that this does not have the same implications in mediation studies as in studies evaluating the effectiveness of an intervention. Since it is the indirect effect of treatment that is evaluated in mediation analysis, the data should represent subjects who have undergone treatment, not necessarily all subjects who started treatment. The results apply to persons who have undergone ACT in a manual-based self-help format with telephone support although data suggest that the format is comparable to face-to-face treatment [36–38].

In the physical functioning scale, respondents are asked to what extent they can carry out routine physical activities with or without pain. From an ACT perspective, the level of pain during an activity is not as relevant as a person’s willingness to perform the activity regardless of pain being present or not. For persons impaired by chronic pain, physical functioning and pain intensity are interconnected but it is important to be aware of the distinction between these two when interpreting the results of the scale.

Although the body of research is continuously growing, the ACT model is still to be investigated further and the mediating effect of all processes in the model should be addressed. It could be argued that the central target of the model is psychological flexibility, hence *that* being the mediating process and that any process preceding that is of less importance. On the other hand, the core elements of the ACT model are distinct from one another and more research is needed to investigate the mediating role of all components of the ACT model.

In conclusion, the study adds to the small but growing body of research investigating the indirect effects of ACT and the results tentatively support the role of acceptance as a mediating variable in the treatment of chronic pain. These findings, however, need to be replicated in future studies.
